# Stripe rust and leaf rust resistance in CIMMYT wheat line “Mucuy” is conferred by combinations of race-specific and adult-plant resistance loci

**DOI:** 10.3389/fpls.2022.880138

**Published:** 2022-08-19

**Authors:** Demei Liu, Chan Yuan, Ravi P. Singh, Mandeep S. Randhawa, Sridhar Bhavani, Uttam Kumar, Julio Huerta-Espino, Evans Lagudah, Caixia Lan

**Affiliations:** ^1^Qinghai Provincial Key Laboratory of Crop Molecular Breeding, Laboratory for Research and Utilization of Qinghai Tibet Plateau Germplasm Resources, Northwest Institute of Plateau Biology, Innovation Academy for Seed Design Chinese Academy of Sciences (CAS), Xining, China; ^2^Hongshan Laboratory, College of Plant Science and Technology, Huazhong Agricultural University, Wuhan, China; ^3^International Maize and Wheat Improvement Center (CIMMYT), Texcoco, Mexico; ^4^International Maize and Wheat Improvement Center (CIMMYT), Nairobi, Kenya; ^5^Borlaug Institute for South Asia (BISA), New Delhi, India; ^6^Campo Experimental Valle de México, Instituto Nacional de Investigacion Forestales Agricolas y Pecuarias (INIFAP), Texcoco, Mexico; ^7^Commonwealth Scientific and Industrial Research Organization (CSIRO) Plant Industry, Canberra, ACT, Australia

**Keywords:** co-located resistance loci, *Puccinia striiformis* f. sp. *tritici*, *P. triticina*, QTL, adult plant resistance

## Abstract

Developing wheat varieties with durable resistance is a core objective of the International Maize and Wheat Improvement Center (CIMMYT) and many other breeding programs worldwide. The CIMMYT advanced wheat line “Mucuy” displayed high levels of resistance to stripe rust (YR) and leaf rust (LR) in field evaluations in Mexico and several other countries. To determine the genetic basis of YR and LR resistance, 138 F_5_ recombinant inbred lines (RILs) derived from the cross of Apav#1× Mucuy were phenotyped for YR responses from 2015 to 2020 at field sites in India, Kenya, and Mexico, and LR in Mexico. Seedling phenotyping for YR and LR responses was conducted in the greenhouse in Mexico using the same predominant races as in field trials. Using 12,681 polymorphic molecular markers from the DArT, SNP, and SSR genotyping platforms, we constructed genetic linkage maps and QTL analyses that detected seven YR and four LR resistance loci. Among these, a co-located YR/LR resistance loci was identified as *Yr29/Lr46*, and a seedling stripe rust resistance gene *YrMu* was mapped on the 2AS/2NS translocation. This fragment also conferred moderate adult plant resistance (APR) under all Mexican field environments and in one season in Kenya. Field trial phenotyping with *Lr37*-virulent *Puccinia triticina* races indicated the presence of an APR QTL accounting for 18.3–25.5% of the LR severity variation, in addition to a novel YR resistance QTL, *QYr.cim-3DS*, derived from Mucuy. We developed breeder-friendly KASP and indel molecular markers respectively for *Yr29*/*Lr46* and *YrMu*. The current study validated the presence of known genes and identified new resistance loci, a QTL combination effect, and flanking markers to facilitate accelerated breeding for genetically complex, durable rust resistance.

## Introduction

Wheat (*Triticum aestivum* L.) is grown on about 215 M ha globally and stands as an indispensable staple food for over 7.5 billion people and an important source of daily calories and protein (http://www.fao.org/home/en/). Biotic stresses, particularly diseases such as wheat rusts, significantly reduce crop yields and quality, particularly where varieties are susceptible and favorable conditions exist. Stripe rust (also known as yellow rust, YR) and leaf rust (LR), caused by *Puccinia striiformis* f. sp. *tritici* (*Pst*) and *P. triticina* (*Pt*), respectively, can cause total crop loss when an early infection strikes in susceptible varieties (Chen, 2005; Bolton et al., 2008). YR generally occurs in cool and moist environments, whereas LR is more adapted to warmer environments coupled with ideal moisture conditions (Zadoks, 1961), but migrating and evolving YR races have infected wheat crops in previously unaffected areas (Ali et al., 2014; Hovmøller et al., 2016). The rusts can be curtailed using fungicides and other measures, but the best control is to grow wheat varieties that carry genetically complex and thus durable disease resistance.

There are three common rust resistance mechanisms in wheat, depending on the host response, the crop growth stage at which the mechanism activates, and the type of resistance gene: (1) race-specific seedling/all-stage resistance, (2) race-specific adult plant resistance (APR), and (3) race non-specific APR (Lan et al., 2017a). The race-specific genes (R-genes) governing seedling/all-stage or adult plant resistance can provide relatively high resistance and are thus easier to select for in breeding. However, given the rapid evolution of the pathogen, R-genes tend to succumb quickly to new rust races, especially when deployed singly, resulting in “boom and bust” cycles of high productivity followed by widespread and potentially disastrous disease outbreaks. In contrast, race-nonspecific APR genes confer partial but broad-spectrum resistance against multiple rust races (Kolmer, 1996) and, when deployed in combinations in wheat varieties, can present a genetically complex resistance that pathogen mutations will not readily overcome. So far, 83 YR and 80 LR resistance genes have been cataloged in wheat (McIntosh et al., 2017; Kumar et al., 2021). Most of these are R-genes, against several of which virulence already exists in the pathogen population. But three pleiotropic multi-pathogen APR genes, *Lr34/Yr18/Sr57/Pm38/Ltn1* (Singh et al., 2012), *Lr67/Yr46/Sr55/Pm46/Ltn3* (Herrera-Foessel et al., 2014), and *Lr46/Yr29/Sr58/Pm39/Ltn2* (Singh et al., 1998), confer partial resistance to LR, YR, stem rust (SR), and powdery mildew (PM) diseases. The first two genes have already been cloned and characterized (Krattinger et al., 2009; Moore et al., 2015), These pleiotropic genes condition partial levels of resistance and a combination of 4–5 APR genes can result in near-immune levels of resistance to rust diseases in CIMMYT wheat germplasm (Singh et al., 2000a).

High-throughput genotyping platforms provide dense coverage of markers, which have enabled the identification of molecular markers linked to resistance genes that are now widely used in wheat breeding programs (Chen, 2013; Rosewarne et al., 2013). Over the last two decades, more than 200 quantitative trait loci (QTL) for YR and LR resistance have been mapped on the 21 wheat chromosomes using diversity arrays technology (DArT), single sequence repeats (SSRs), and single-nucleotide polymorphisms (SNP) marker platforms (McIntosh et al., 2017). In addition, 11 potential co-located APR QTL conferring pleiotropic resistance to YR, LR, and PM on chromosomes 1BS, 1BL, 2AL, 2BS (two QTL), 2DL, 4DL, 5BL, 6AL,7BL, and 7DS were identified through comparative mapping (Li et al., 2014).

When distributed for international testing in 2013, the advanced CIMMYT breeding line “Mucuy” showed high levels of resistance to both YR and LR in several countries. Mucuy was released in 2017 as “Super 272” in the Northwestern Plain Zone of India, where YR is prevalent. The line also resisted YR races in Kenya and Ethiopia, where PstS2 and PstS11 are the predominant race groups, prompting further studies to understand the genetics of rust resistance in Mucuy. The current study sought to (1) investigate the genetic bases of YR and LR resistance using an F_5_ recombinant inbred line (RIL) population derived from the cross of “Apav#1” × “Mucuy;” (2) identify loci conferring resistance at both the seedling and adult plant stages using molecular markers; and (3) understand the QTL combinations effects on YR and LR, among identified resistance loci.

## Materials and methods

### Plant materials

We used 138 F_4_-derived F_5_ RILs from the cross of Apav#1 × Mucuy. The susceptible parent, Apav#1 (CIMMYT Germplasm ID: 1853706), derived from a cross of “Avocet-*YrA*” × “Pavon 76,” was susceptible to YR, LR, and stem rust (SR) at all growth stages, against predominant *Pst* and *Pt* races used in various trials in Mexico. In contrast, Mucuy (CIMMYT Germplasm ID: 5663955), derived from the cross “Mutus”^*^2 × “Akuri,” showed intermediate resistance during seedling evaluations in the greenhouse but high resistance to both YR and LR at the adult plant stage in field trials, in both cases against races *Pst* and *Pt*. The RILs were developed following a bulk advancement of the population until the F_4_ generation and then harvesting random plants individually to obtain F_5_ RILs (Basnet et al., 2014a). We multiplied the RIL seed and used it in all studies.

### Seedling evaluations

#### Stripe rust

For YR seedling evaluations, the parents and F_5_ RILs were grown in a greenhouse, and seedlings inoculated at the two-leaf stage with *Pst* isolate Mex14.191 (Avirulence/virulence: *Yr1, 4, 5a, 10, 15, 24, 26, 5b, Poll*/*Yr2, 3, 6, 7, 8, 9, 17, 27, 31, A*; Randhawa et al., 2018). A set of 30 differential lines possessing the known YR resistance genes, mostly in the Avocet background, were also included. An atomizer was used to spray urediniospores suspended in light-weight mineral oil Soltrol 170 (Chempoint.com) at the two-leaf stage. Inoculated plants were moved to a dew chamber at 7°C for 24 h after mineral oil had evaporated from the leaf surface to facilitate spore germination and initiate infection, and then transferred back to greenhouse benches for disease development. The minimum, maximum, and average temperatures of the greenhouse were 9.4, 20.8, and 16.1°C. Infection type (IT) data was recorded 2 weeks post-inoculation using the modified 0-9 scale (McNeal et al., 1971), where 0 = no visible infection, 1 = necrotic/chlorotic flecks without sporulation, 2 = necrotic/chlorotic stripes without sporulation, 3 = necrotic/chlorotic stripes with trace sporulation, 4 = necrotic/chlorotic stripes with light sporulation, 5 = necrotic/chlorotic stripes with intermediate sporulation, 6 = chlorosis stripes with abundant sporulation, 7 = chlorotic stripes with abundant sporulation, 8 = stripe without chlorosis, moderate sporulation, 9 = stripes without chlorosis and abundant sporulation. Infection types “7,” “8,” and “9” were considered susceptible; all others were recorded as resistant.

#### Leaf rust

Parents and RILs were evaluated at seedling stage with *Pt* races MBJ/SP (isolate MEX96.560) [Avirulence/virulence: *Lr2a, 2b, 2c, 3ka, 9, 16, 19, 21, 24, 25, 28, 29, 30, 32, 33, 36*/*1, 3, 3bg, 10, 11, 12, 13, 14a, 14b, 15, 17a, 18, 20, 23*, (*26*), *27*+*31, 37*; Herrera-Foessel et al., 2012; Huerta-Espino et al., 2020] and TBD/TM [isolate MEX91.28A; Avirulence/virulence: *Lr3ka, 11, 16*, (*23*), *24, 26, 37/1, 2a, 2b, 2c, 3, 3bg, 10, 13, 14a, 15, 17, 18, 27*+*31, 28*; Singh, 1991]. The latter race was used for better expression of *Lr16* resistance, postulated to be present in Mucuy and segregated in the RIL population. A set of 48 lines with known LR genes, mostly in the “Thatcher” background, were also included. Inoculation was performed as for YR but with overnight misting at room temperatures and the minimum, maximum, and average temperatures were maintained at 9.0, 23.0, and 18.1°C, for disease development with both *Pt* races. LR ITs were recorded 11 days post-inoculation using the 0–4 scale (Roelfs et al., 1992), where 0 = no visible symptoms,; = necrotic/chlorotic flecks, 1 = small uredinia surrounded by necrosis, 2 = small-to-medium uredinia surrounded by chlorosis or necrosis, X = random distribution of variable-sized uredinia, 3 = medium-sized uredinia without chlorosis, 4 = large uredinia without chlorosis, and + and – indicated a bit larger or smaller uredinia than normal for the infection type. Infection types “3” and “4” were considered susceptible while all other infection types were considered resistant.

### Field experiments

#### Stripe rust

We conducted YR field evaluations at the CIMMYT research station at Toluca, State of Mexico, Mexico, during the 2015, 2016, and 2017 seasons (hereafter referred to as YrMV15, YrMV16, and YrMV17), at Kenya Agriculture and Livestock Research Organization (KALRO) research station in Njoro, Kenya, during the main-season of 2016 and off-seasons of 2016, 2019, and 2020 (referred as YrKE16M, YrKE16O, YrKE19, and YrKE20), and at the research station of Borlaug Institute for South Asia (BISA) in Ludhiana, India, during the 2018–2019 and 2019–2020 seasons (referred as YrIN19 and YrIN20). Each genotype was sown in 0.7-m paired rows with a 0.3-m pathway between rows. Field trials were unreplicated, given rust resistance's highly heritability when phenotyping is conducted under managed epidemics at hot spot sites where disease pressure is maximum. At Toluca, the YR spreader rows consisted of a mixture of susceptible wheat lines (*Yr27*-carrying lines derived from the “Avocet” × “Attila” cross, “Morocco” and “Avocet+*Yr31*”). This spreader mixture was planted both as hill plots in the middle of the 0.3-m pathway and around the experimental nursery. The same *Pst* isolate (Mex14.191) used for seedling evaluations was sprayed onto YR spreaders about 4 weeks post-germination and this was repeated three times to initiate artificial epidemics. At the KALRO station, YR evaluations were carried out under natural epidemics and the causal race was identified as PstS11 [Avirulence/virulence: *Yr1, 3, 5, 9, 10, 15, 24, 25, 26, Sp, Amb/2, (4), 6, 7, 8, 17, 27, 32, AvS*] by the GRRC (Global Rust Reference Center), Denmark. Inoculation at Ludhiana was carried out using spreader rows of the variety PBW343, known to carry *Yr27*, inoculated with a mixture of races 110S84, 46S119, 110S119, and 238S119 (Supplementary Table 1) that are predominant in this region. Even though the inoculated races dominate in the screening nurseries, the presence of natural inoculum carrying other races at low frequencies cannot be ruled out. The key difference in *Pst* populations in Mexico vs. Kenya and India is the avirulence for resistance gene *Yr4* in Mexico but virulence for it in the races of Kenya and India, based on the response of the “Avocet+*Yr4*” tester line.

#### Leaf rust

In the 2015–2016, 2016–2017, and 2017–2018 growing seasons, the parents and RILs were evaluated for APR to LR at Norman E. Borlaug Research Station (CENEB), Ciudad Obregón, State of Sonora, Mexico (hereafter referred to as LrY16, LrY17, and LrY18). The field experiment design was similar to that for YR. The susceptible LR spreader lines included Morocco and Avocet near-isolines carrying *Yr24/26*. The mixture of *Pt* races MCJ/SP [isolate MEX94.47; Avirulence/virulence: *Lr2a, 2b, 2c, 3ka, 9, 16, 19, 21, 24, 25, 28, 29, 30, 32, 33, 36/1, (3), 3bg, 10, 11, 12, 13, 14a, 14b, 15, 17a, 18, 20, 23, 26, 27*+*31, 37*; Herrera-Foessel et al., 2012] and MBJ/SP (isolate MEX96.560, same as MCJ/SP except virulent on *Lr3* and avirulent on *Lr26*) was suspended in Soltrol 170 and sprayed on the spreaders to cause artificial epidemics.

### Disease severity evaluation and statistical analyses

Disease severity (DS) of the parents and RILs were recorded on 3 occasions using the modified Cobb's Scale (Peterson et al., 1948). Initial data were recorded when the DS of Apav#1 was around 80% and repeated after 7 days. The last data set was recorded when it reached 100%. For multiple disease readings, the area under the disease progress curve (AUDPC) was calculated as per Bjarko and Line (1988). The correlation analysis of final disease severity (FDS) in each environment was conducted using SAS 9.4 software (SAS Institute, Cary, NC).

### Genetic linkage map construction and QTL mapping

DNA was extracted using the CTAB method (Dreisigacker et al., 2012) from one-week-old seedlings of the parents and RILs grown in a greenhouse and genotyped with the DArT-GBS platform (Reference for SAGA). In total, we genotyped 40,519 GBS-in-Silico and 39,849 GBS-SNP, in combination with closely linked molecular markers for different rust genes (*Xgwm210* for *Lr16, cslv46G22* for *Lr46/Yr2*9, two *Yr17*-linked markers *WGGB156* and *WGGB159* by Wang et al. (2018), and one *Yr17*-linked marker provided by Evans Lagudah), in the entire RIL population. Genetic linkage maps were constructed using Joinmap 4.1 (Van Ooijen, 2006) with 12,681 polymorphic markers, and 56 linkage groups were constructed. In addition, QTL analysis was performed by inclusive composite interval mapping (ICIM) using IciMapping 4.2 (Meng et al., 2015) with the DS of each tested environment and the mean of FDS (referred to as YrM for YR and LrM for LR). The logarithm of odds (LOD) score was determined based on the 1,000 permutation test and a significance level of α = 0.05. The percentages of phenotypic variance explained (PVE) were determined using stepwise regression at the LOD peaks (Somers et al., 2004; Francki et al., 2009; Huang et al., 2012; Wilkinson et al., 2012).

## Results

### Seedling responses

#### Stripe rust

**Figure 1:**
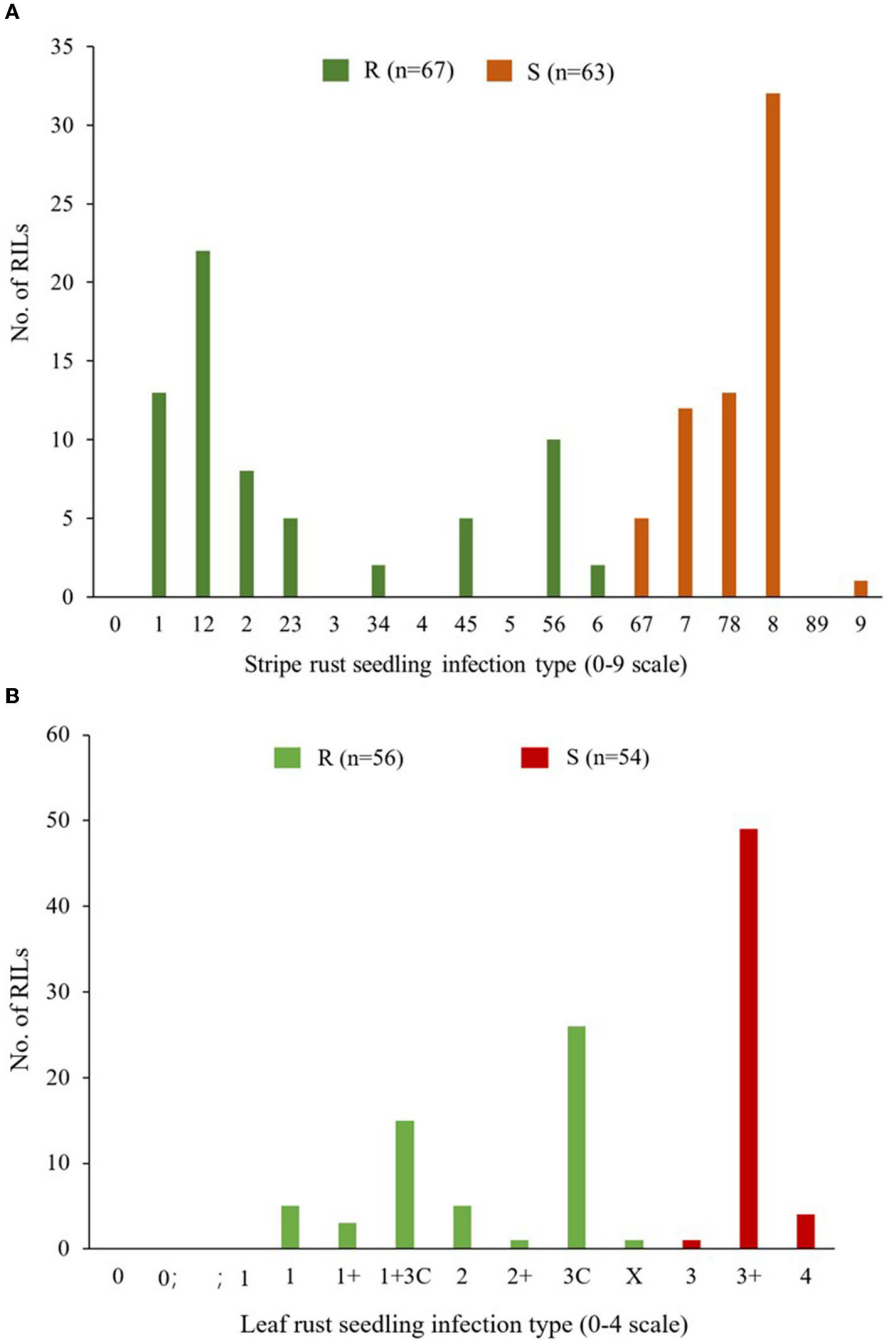
Frequency distributions of the Apav#1×Mucuy recombinant inbred lines (RILs) for stripe rust (YR) responses **(A)** and leaf rust (LR) responses **(B)** grouped as resistant (R) and susceptible (S) at the seedling stage. For stripe rust, IF ≤ 6 is in the R group, while the rest lines with IF ≥ 67 are in the S group. For leaf rust, IF ≤ X is the R group and IF ≥ 3 is the S group.

Apav#1 and Mucuy showed seedling infection type (IT) responses of “8” and “1,” respectively, against *Pst* isolate Mex14.191. Seedling evaluation of the RILs identified 67 resistant lines (ITs ranging from 1 to 6) and 63 susceptible lines (ITs ranging from 67 to 9); segregating lines were excluded from the analysis (Figure 1A). *Chi*-squared analysis of goodness of fit suggested segregation of a single resistance gene in this population (χ^2^ = 0.069, *P* = 0.73). It was temporarily named as *YrMu*, mapped on wheat chromosome 2AS at 16.6–19.1 Mb (International Wheat Genome Sequencing Consortium, 2018), and co-segregated with 79 molecular markers including *WGGB156, WGGB159*, and *InD_hzau_MuYLr-2AS* (Supplementary Figure 1A). *WGGB156* and *WGGB159* had previously been confirmed as closely linked to *Yr17* (Wang et al., 2018). In addition, we phenotyped the Avocet+*Yr17* isoline against Mex14.191 and the IT response was “8.” Thus, we speculated that *YrMu* might be a new stripe rust resistance gene in Mucuy at the seedling stage against Mex14.191, although the possibility of an enhanced expression of *Yr17* due to the genetic background cannot be ruled out, due to the continuous variation for resistance phenotypes included in the resistance category.

#### Leaf rust

Seedling evaluation of parents under LR showed IT responses “4” for Apav#1 and “3C” for Mucuy, against the *Pt* race MBJ*/*SP. The distribution of 56 resistant and 54 susceptible RILs conformed to the segregation of a single resistance gene (χ^2^ = 0.009, *P* = 0.85) that mapped on the short arm of chromosome 2B in the interval of molecular markers *2325486* and *4405950* (Figure 1B, Supplementary Figure 1B). The infection types of Mucuy and resistant RILs were similar to the tester line for gene *Lr16*, hence this gene might be *Lr16*.

### Adult plant response

**Figure 2:**
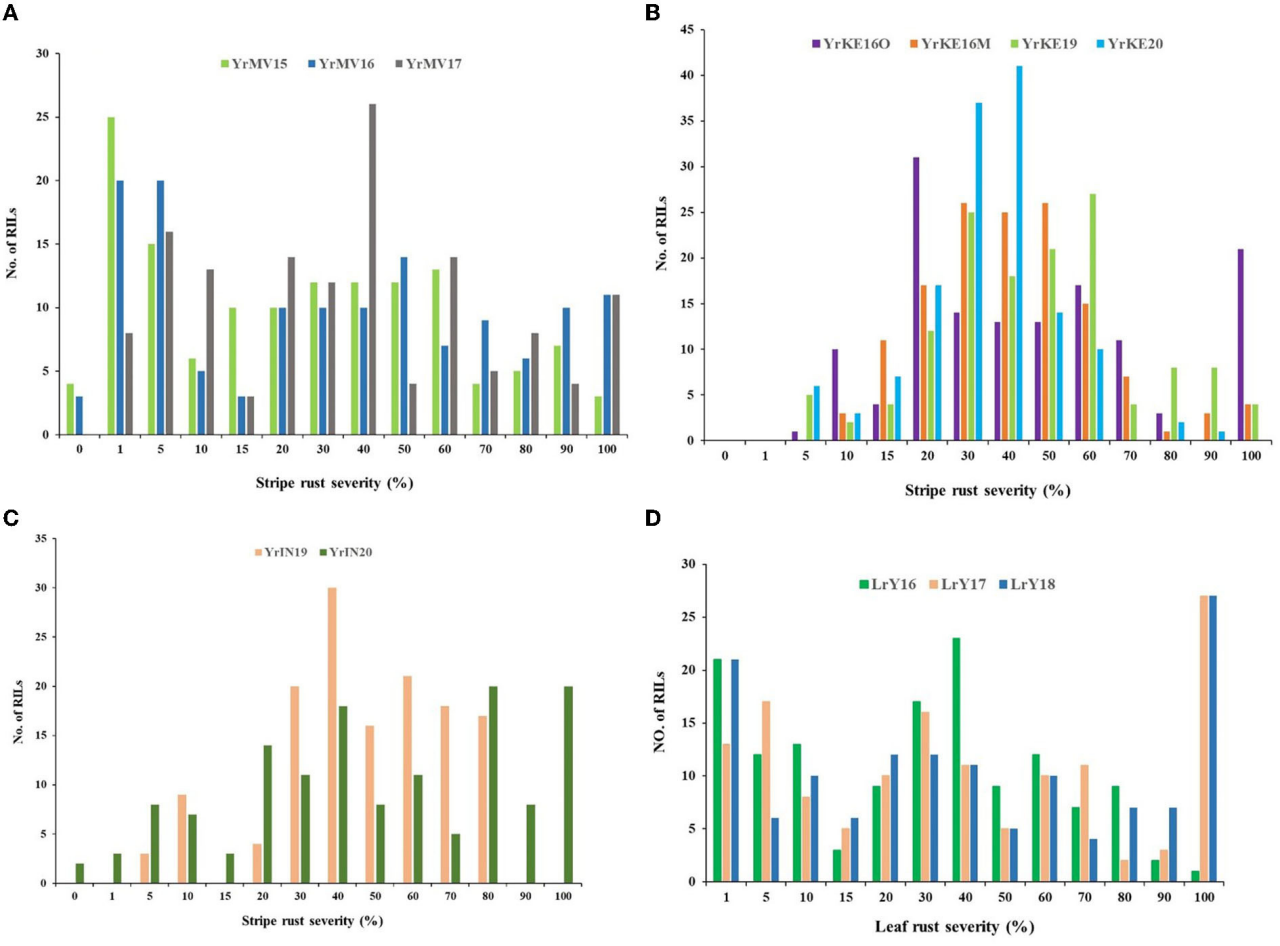
Frequency distributions of Apav#1×Mucuy recombinant inbred lines (RILs) for the final stripe rust severities in three environments in Mexico **(A)**, four environments in Kenya **(B)**, and two environments in India **(C)**, along with final leaf rust severities in three environments in Mexico **(D)**.

The FDS and host response to YR for Apav#1 ranged from 70 to 100 S and for Mucuy ranged from 0 to 5 MS at the adult plant stage over 9 environments. The continuous distribution of YR DS for RILs in each environment indicated the polygenic inheritance of APR (Figures 2A–C).

LR FDS and host responses were 100 S for Apav#1 and 0 for Mucuy over all 3 seasons. The LR DS frequency distributions were distorted and skewed toward the resistance (Figure 2D), indicating the segregation of at least one large-effect LR resistance locus in the population.

### Correlation coefficients

The correlation coefficients of FDS for RILs varied from 0.23 to 0.93 in the nine YR environments (Table 1), while the phenotypic correlation coefficient was 0.90 to 0.93 across the three Mexican environments. Low phenotypic correlation coefficients among Mexico, Kenya, and India were attributed to the presence of different rust isolates in these locations. For LR FDS, the correlation coefficients among the three test environments in Mexico were high, ranging from 0.86 to 0.89 (Table 1). In addition, significant phenotypic correlations, ranging from 0.30 to 0.75, occurred in all environments between LR and YR (Table 1), indicating the presence of pleiotropic/co-located resistance loci in the population.

**Table 1:** Phenotypic correlations among final stripe rust severities in nine environments (Toluca YrMV15, YrMV16, and YrMV17; Kenya YrKE16O, YrKE16M, YrKE19, YrKE20; India YrIN19, YrIN20) and final leaf rust severities in three environments (Ciudad Obregón LrY16, LrY17, and LrY18), all in Mexico.

**Environment**	**YrMV15**	**YrMV16**	**YrMV17**	**YrKE16O**	**YrKE16M**	**YrKE19**	**YrKE20**	**YrIN19**	**YrIN20**	**LrY16**	**LrY17**
YrMV16	0.93**										
YrMV17	0.90**	0.91**									
YrKE16O	0.42**	0.47**	0.42**								
YrKE16M	0.70**	0.73**	0.73**	0.51**							
YrKE19	0.23**	0.29**	0.34**	0.23**	0.53**						
YrKE20	0.26**	0.31**	0.34**	0.26**	0.51**	0.76**					
YrIN19	0.34**	0.37**	0.37**	0.44**	0.47**	0.30**	0.37**				
YrIN20	0.52**	0.54**	0.54**	0.36**	0.57**	0.33**	0.36**	0.44**			
LrY16	0.68**	0.74**	0.72**	0.47**	0.67**	0.41**	0.38**	0.32**	0.43**		
LrY17	0.64**	0.70**	0.67**	0.46**	0.69**	0.43**	0.37**	0.28**	0.47**	0.88**	
LrY18	0.60**	0.67**	0.64**	0.49**	0.65**	0.38**	0.33**	0.31**	0.42**	0.86**	0.89**

^**^P < 0.001.

### Co-located resistance loci

**Figure 3:**
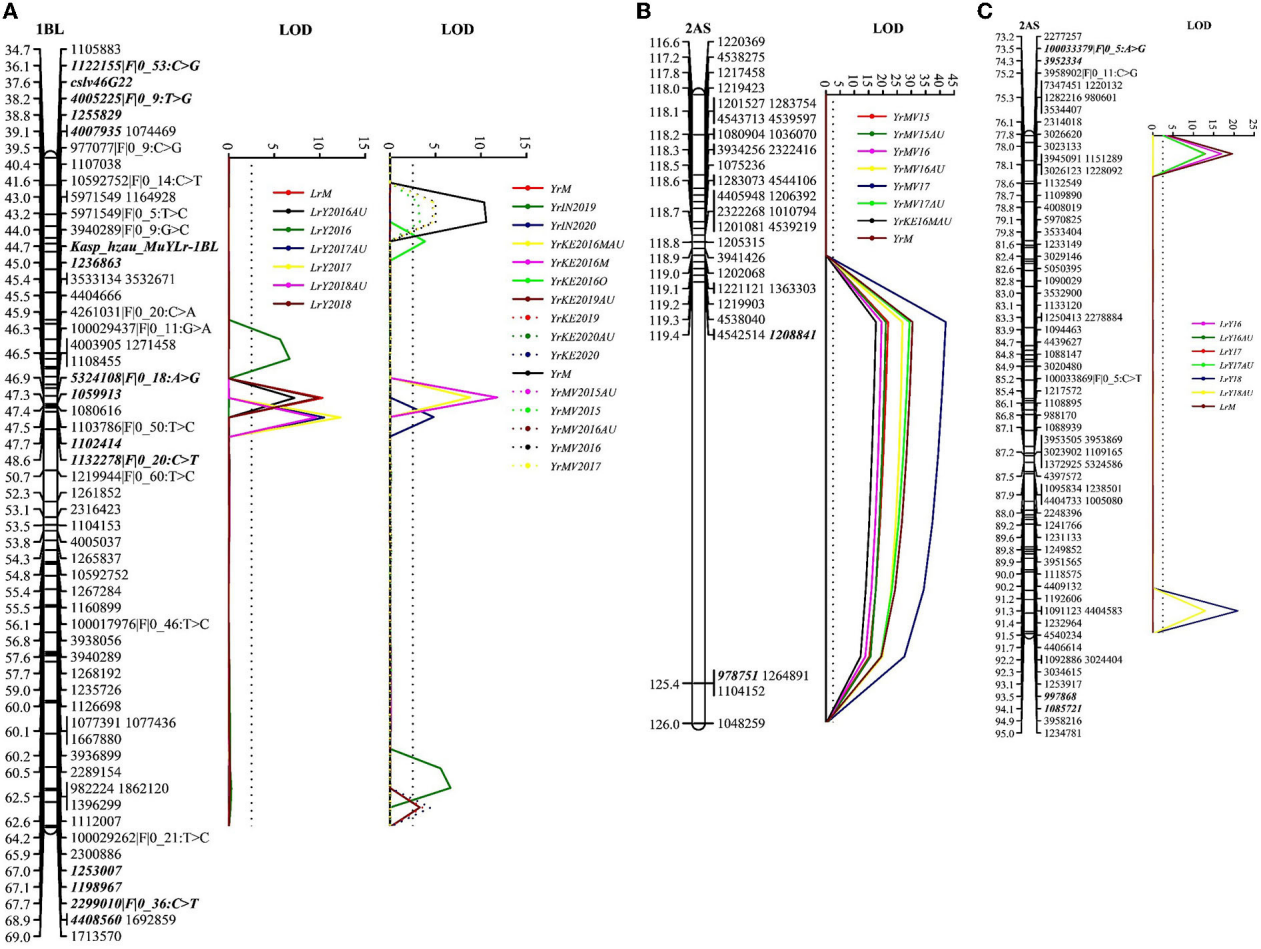
The logarithm of odds (LOD) plot of quantitative trait loci (QTL) for adult plant resistance to both stripe rust and leaf rust on chromosomes 1BL **(A)**, 2AS for YR **(B)**, and 2AS for LR **(C)** in the Apav#1×Mucuy RIL population. Positions (cM) of the molecular markers on chromosomes are shown on the vertical axes; cumulative genetic distances of linkage groups are also shown. QTL flanking markers are in bold.

We identified two co-located resistance loci for YR and LR in the RIL population. The first locus was located on chromosome 1BL and designated *QYr.cim-1BL/QLr.cim-1BL*. This locus was detected in all tested YR and LR environments and accounted for 10.4–33.3% of YR phenotypic variation and 20.6–33.6% of LR phenotypic variation (Table 2, Figure 3A). Based on the closely linked molecular markers, we developed a KASP marker, such as *Kasp_hzau_MuYLr-1BL*, and genotyped the entire RIL population (Supplementary Figure 2, Supplementary Table 2). The single marker analysis showed highly significant mean differences of both YR and LR for RILs carrying the positive allele and those lacking it (Supplementary Table 3). *Kasp_hzau_MuYLr-1BL* was one of the flanking markers of *QLr.cim-1BL* (Table 2). Because the known pleiotropic multi-pathogen slow-rusting resistance gene *Lr46/Yr29/Sr58/Pm39* is also located on 1BL, we genotyped the RIL population with the closely linked molecular marker *cslv46G22*. The result showed that *cslv46G22* was one of the flanking markers of *QYr.cim-1BL/QLr.cim-1BL* as well.

**Table 2:** Position and effects of quantitative trait loci (QTL) for adult plant resistance (APR) to stripe rust, leaf rust, and mean of final stripe and leaf rust severities over all tested environments (YrM and LrM), using inclusive composite interval mapping (ICIM) by IciMapping 4.2 in the 138 Apav#1 × Mucuy F_5_ RIL population.

**QTL^a^**	**Trait name**	**Position^b^**	**Left marker**	**Right marker**	**Physical position^c^**	**LOD^d^**	**PVE (%)^e^**	**Add^f^**
*QLr.cim-1BL*	LrY16	45	Kasp_hzau_MuYLr-1BL	1236863	669.2–670.5	6.7	20.6	5.3
*(Lr46*)	LrY16AU^g^	47	5324108|F|0_18:A>G	1059913	669.0–669.2	7.2	22.0	110.6
	LrM	47	5324108|F|0_18:A>G	1059913	669.0–669.2	10.3	29.3	17.1
	LrY18	47	5324108|F|0_18:A>G	1059913	669.0–669.2	9.8	28.2	17.8
	LrY17AU	48	1102414	1132278|F|0_20:C>T	669.2–669.8	10.5	29.5	193.6
	LrY17	48	1102414	1132278|F|0_20:C>T	669.2–670.5	12.3	33.6	20.7
	LrY18AU	48	1102414	1132278|F|0_20:C>T	669.2–670.5	9.6	27.4	125.4
*QLr.cim−2AS*	LrY16	74	100033379|F|0_5:A>G	3952334	4.9–19.4	16.9	24.8	13.9
*(2NS)*	LrY16AU	74	100033379|F|0_5:A>G	3952334	4.9–19.4	19.4	25.5	129.6
	LrY17	74	100033379|F|0_5:A>G	3952334	4.9–19.4	12.9	20.3	15.3
	LrY17AU	74	100033379|F|0_5:A>G	3952334	4.9–19.4	12.8	21.3	143.0
	LrY18	94	997868	1085721	15.3–15.4	20.9	24.2	17.3
	LrY18AU	94	997868	1085721	15.3–15.4	12.9	18.3	101.6
	LrM	74	100033379|F|0_5:A>G	3952334	4.9–19.4	19.6	19.6	14.7
*QLr.cim-2BS*	LrY16	183	4989699	1016414	14.4–17.4	9.9	9.6	10.8
*(Lr16)*	LrY16AU	183	4989699	1016414	14.4–17.4	7.8	9.0	7.3
	LrY17	203	1100485|F|0_14:C>T	1224458	13.9–23.9	11.9	13.1	91.5
	LrY17AU	204	1224458	1126406|F|0_59:A>G	23.9	9.5	17.5	8.7
	LrY18	203	1100485|F|0_14:C>T	1224458	13.9–23.9	8.1	11.7	11.5
	LrY18AU	203	1100485|F|0_14:C>T	1224458	13.9–23.9	10.9	15.8	93.3
	LrM	203	1100485|F|0_14:C>T	1224458	13.9–23.9	9.7	12.0	9.5
*QLr.cim-5AL*	LrY16	170	1204040|F|0_64:G>C	1141498|F|0_63:T>C	585.2–589.2	5.6	6.0	3.1
	LrY16AU	170	1204040|F|0_64:G>C	1141498|F|0_63:T>C	585.2–589.2	6.1	6.9	64.9
	LrY18	170	1204040|F|0_64:G>C	1141498|F|0_63:T>C	585.2–589.2	5.3	6.5	9.7
	LrY18AU	170	1204040|F|0_64:G>C	1141498|F|0_63:T>C	585.2–589.2	5.9	4.8	57.5
	LrM	170	1204040|F|0_64:G>C	1141498|F|0_63:T>C	585.2–589.2	4.9	4.7	7.2
*QYr.cim-1AL*	YrMV15	201	1125323|F|0_58:T>G	2259648|F|0_5:A>G	578.3–580.0	4.8	5.4	−5.4
	YrMV16AU	181	1101176	4989882	578.2–579.0	4.0	2.2	−45.5
	YrKE16M	213	3064615|F|0_6:C>T	996385|F|0_7:C>G	578.7–579.3	5.4	3.8	−4.0
	YrKE19	169	989816|F|0_24:A>C	1265000	575.2–577.9	16.5	14.6	−6.1
	YrKE19AU	167	987869|F|0_42:A>C	989816|F|0_24:A>C	565.5–575.2	8.9	6.6	−69.4
	YrM	162	987869|F|0_42:A>C	989816|F|0_24:A>C	565.5–575.2	7.8	4.4	−4.5
*QYr.cim-1BL*	YrMV16AU	37	1122155|F|0_53:C>G	cslv46G22	670.2–670.4	4.7	15.8	143.9
*(Yr29)*	YrM	38	cslv46G22	4005225|F|0_9:T>G	669.9–670.2	10.6	30.1	11.1
	YrMV15	38	cslv46G22	4005225|F|0_9:T>G	669.9–670.2	3.3	10.4	8.5
	YrMV15AU	38	cslv46G22	4005225|F|0_9:T>G	669.9–670.2	3.3	10.5	61.8
	YrMV16	38	cslv46G22	4005225|F|0_9:T>G	669.9–670.2	5.0	16.2	13.3
	YrMV17	38	cslv46G22	4005225|F|0_9:T>G	669.9–670.2	4.8	15.0	11.6
	YrKE16O	39	1255829	4007935	670.5–671.6	3.9	12.3	22.1
	YrKE16M	47	5324108|F|0_18:A>G	1059913	669.0–669.2	11.8	33.3	11.5
	YrKE16MAU	47	5324108|F|0_18:A>G	1059913	669.0–669.2	8.8	26.6	125.3
	YrIN20	48	1102414	1132278|F|0_20:C>T	669.2–669.8	4.8	14.8	12.5
	YrIN19	67	1253007	1198967	674.5	6.6	19.9	9.3
	YrKE19	68	2299010|F|0_36:C>T	4408560	673.5	3.6	11.3	7.9
	YrKE19AU	68	2299010|F|0_36:C>T	4408560	673.5	3.3	10.4	87.7
	YrKE20	68	2299010|F|0_36:C>T	4408560	673.5	4.5	14.3	5.8
	YrKE20AU	68	2299010|F|0_36:C>T	4408560	673.5	4.5	14.2	32.0
*QYr.cim-2AS*	YrMV15	120	1208841	978751	31.9–32.8	21.8	32.3	13.2
*(YrMu)*	YrMV15AU	120	1208841	978751	31.9–32.8	21.1	26.5	79.0
	YrMV16	120	1208841	978751	31.9–32.8	19.6	24.7	13.7
	YrMV16AU	120	1208841	978751	31.9–32.8	26.9	23.3	146.9
	YrMV17	120	1208841	978751	31.9–32.8	42.1	54.1	22.8
	YrMV17AU	120	1208841	978751	31.9–32.8	29.4	31.3	188.4
	YrKE16MAU	120	1208841	978751	31.9–32.8	17.6	28.1	127.6
	YrM	120	1208841	978751	31.9–32.8	30.4	26.2	11.1
*QYr.cim-3AS*	YrMV15	266	4010188	1092360	–	6.0	3.7	4.2
	YrMV16	250	4989420	1140071	9.4–10.2	4.3	4.5	3.9
	YrKE16M	235	1139244	3937315	6.6–7.9	4.4	3.7	2.1
	YrKE20	229	4990593	3951957	8.2	5.5	7.1	4.3
	YrKE20AU	242	4398142	4536273	8.5	12.3	12.6	35.0
	YrM	243	4536273	4009657	8.5–8.7	6.8	3.8	4.2
*QYr.cim-3BS*	YrMV16	610	1051249|F|0_64:T>G	1128851|F|0_5:C>T	24.9	4.8	5.1	6.3
	YrMV16AU	613	1051249|F|0_64:T>G	1128851|F|0_5:C>T	24.9	7.5	4.8	66.5
	YrMV17	621	1056536|F|0_58:A>G	1004919|F|0_40:G>C	24.8–24.9	5.2	3.3	5.6
	YrIN19	797	1315407|F|0_8:G>A	1318182	10.6–10.9	8.5	10.7	8.1
	YrIN20	722	1109710|F|0_29:C>T	1076654|F|0_12:T>C	17.8–17.9	4.6	10.2	10.2
*QYr.cim-3DS*	YrKE16M	26	1091508	1215873	85.0–90.7	4.7	3.6	3.9
	YrKE19	37	2261207	1143346	78.7–81.7	9.6	7.7	6.4
	YrKE19AU	37	2261207	1143346	78.7–81.7	9.5	7.1	72.8
*QYr.cim-6BS*	YrMV15	55	2261971|F|0_23:A>C	1000134|F|0_15:T>C	126.1–153.4	4.1	2.5	3.4
	YrMV15AU	67	1128426|F|0_22:C>T	1109468|F|0_15:G>A	115.7–117.3	10.2	10.4	49.4
	YrMV17	193	1239693	1218710	134.7–151.4	4.0	10.1	4.9
	YrKE19	55	2261971|F|0_23:A>C	1000134|F|0_15:T>C	126.1–153.4	6.9	6.8	4.9
	YrKE19AU	53	7353260|F|0_50:A>T	2261971|F|0_23:A>C	126.1–151.9	6.7	4.7	58.3

^a^QTL that extends across single one-log support confidence gaps was assigned the same symbol.

^b^Peak position in centi-Morgans from the first linked marker of the relevant linkage group.

^c^Based on the reference genome of Chinese Spring (CS) (IWGSC).

^d^Logarithm of odds (LOD) score based on 1,000 permutations.

^e^PVE is the proportion of phenotypic variance explained by the QTL.

^f^Additive effect of phenotypic variance for each QTL.

^g^The area under the disease progress curve (AUDPC).

The second QTL was *QYr.cim-2AS/QLr.cim-2AS*, located on the short arm of chromosome 2A and which explained 23.3–54.1 and 18.3–25.5% of YR and LR DS variations, respectively. *QYr.cim-2AS* was detected in all Mexican YR environments and 1 year of the Kenyan environment and was located in the interval of (DArT)-GBS markers *1208841* and *978751* (Table 2, Figure 3) within 2.1 cM from the seedling resistance gene *YrMu*. We also mapped a leaf rust resistance QTL, *QLr.cim-2AS*, on 2AS, which was flanked by molecular markers *100033379|F|0_5:A*>*G, 3952334, 997868* and *1085721* (Table 2, Figure 3C). We developed an InDel marker named *InD_hzau_MuYLr-2AS* that was genotyped on the F_5_ RIL population (Supplementary Table 2); the single marker analysis showed that it was significantly correlated with both YR and LR phenotypes and co-located with *YrMu* (Supplementary Table 4). Thus, the InDel marker *InD_hzau_MuYLr-2AS* can be used in wheat breeding to select for QTL *QYr.cim-2AS/QLr.cim-2AS*.

### Other QTL conferring APR to YR or LR

In addition to the 2 co-located resistance loci mentioned above, we found 4 more resistance loci derived from Mucuy that confer APR to YR, named *QYr.cim-3AS, QYr.cim-3BS, QYr.cim-3DS* and *QYr.cim-6BS*, in combination with a locus, *QYr.cim-1AL*, contributed by Apav#1. Identified at both the Mexican and Kenyan testing locations in 2 years, *QYr.cim-3AS* was associated with molecular markers *4010188, 1092360, 4989420, 1140071, 1139244, 3937315, 4990593, 3951957, 4398142, 4536273*, and *4009657* (Table 2). Its corresponding physical locations on the Chinese Spring (CS) reference genome (International Wheat Genome Sequencing Consortium, 2018) ranged from 6.6 to 10.2 Mb and it explained 3.7–12.6% of the YR phenotypic variation. *QYr.cim-3BS* was in vicinity of the molecular markers *1051249|F|0_64:T*>*G, 1128851|F|0_5:C*>*T, 1056536|F|0_58:A*>*G, 1004919|F|0_40:G*>*C, 1315407|F|0_8:G*>*A, 1318182, 1109710|F|0_29:C*>*T*, and *1076654|F|0_12:T*>*C*, and the corresponding physical locations on the CS reference genome (International Wheat Genome Sequencing Consortium, 2018) spanned from 10.6 to 24.9 Mb. It was detected in 2 years at each of the Mexican and Indian locations and accounted for 3.3–10.7% of YR phenotypic variation (Table 2). *QYr.cim-3DS* was detected in 2 years at the Kenyan location, explained 3.6–7.7% of YR phenotypic variation, and was linked to markers *1091508, 1215873, 2261207*, and *1143346* in physical positions spanning from 78.7 Mb to 90.7 Mb (International Wheat Genome Sequencing Consortium, 2018; Table 2). *QYr.cim-6BS* was located near markers *2261971|F|0_23:A*>*C, 1000134|F|0_15:T*>*C, 1128426|F|022:C*>*T, 1109468|F|0_15:G*>*A, 1239693, 1218710, 7353260|F|0_50:A*>*T* and *2261971|F|0_23:A*>*C*, with physical positions ranging from 115.7 to 153.4 Mb (International Wheat Genome Sequencing Consortium, 2018). *QYr.cim-6BS* was detected for 2 years at the Mexican location and 1 year at the Kenyan location, and it explained 2.5–10.4% of the YR phenotypic variation. *QYr.cim-1AL* for YR resistance was the only locus identified that was derived from Apav#1. It explained 2.2–14.6% of the phenotypic variation, had a physical position ranging from 565.5 to 580.0 Mb (International Wheat Genome Sequencing Consortium, 2018), and was flanked by molecular markers *1125323|F|0_58:T*>*G, 22599648|F|0_5:A*>*G, 1101176, 4989882, 3064615|F|0_6:C*>*T, 996385|F|0_7:C*>*G, 989816|F|0_24:A*>*C, 126500, 987869|F|0_42:A*>*C* and *989816|F|0_24:A*>*C* (Table 2).

We found 2 more resistance loci derived from Mucuy that conferred APR to LR, named *QLr.cim-2BS* and *QLr.cim-5AL*. Both loci were consistently identified in all LR environments except for *QLr.cim-5AL* in LrY17. *QLr.cim-2BS* explained 9.0–17.5% of LR phenotypic variation and was located on the short arm of chromosome 2B. It was flanked by molecular markers *4989699, 1016414, 1100485|F|0_14:C*>*T, 1224458*, and *1126406|F|0_59:A*>*G* (Table 2). The seedling LR resistance gene *Lr16* was also identified on 2BS. The physical location of *Lr16* was 13.7–23.9 Mb, based on the CS reference genome (International Wheat Genome Sequencing Consortium, 2018), meaning it overlapped with *QLr.cim-2BS* at 13.9–23.9 Mb. This confirmed that *QLr.cim-2BS* and *Lr16* should be the same gene that provided all-stage resistance to LR in the RIL population. *QLr.cim-5AL* was located in the interval of molecular markers *1204040|F|0_64:G*>*C* and *1141498|F|0_63:T*>*C* and explained 4.7–6.9% of LR phenotypic variation (Table 2).

### Phenotypic effects of QTL combinations

Due to the differences among rust races in different countries, resistance effects provided by individual loci varied greatly at different locations. For example, *QYr.cim-2AS* provided significant resistance in Mexico, could not be detected in India and was identified in Kenya only in 2016. Therefore, we analyzed the phenotypic effects of QTL combinations among 4 stably detected QTLs according to the average DS in the three countries.

**Figure 4:**
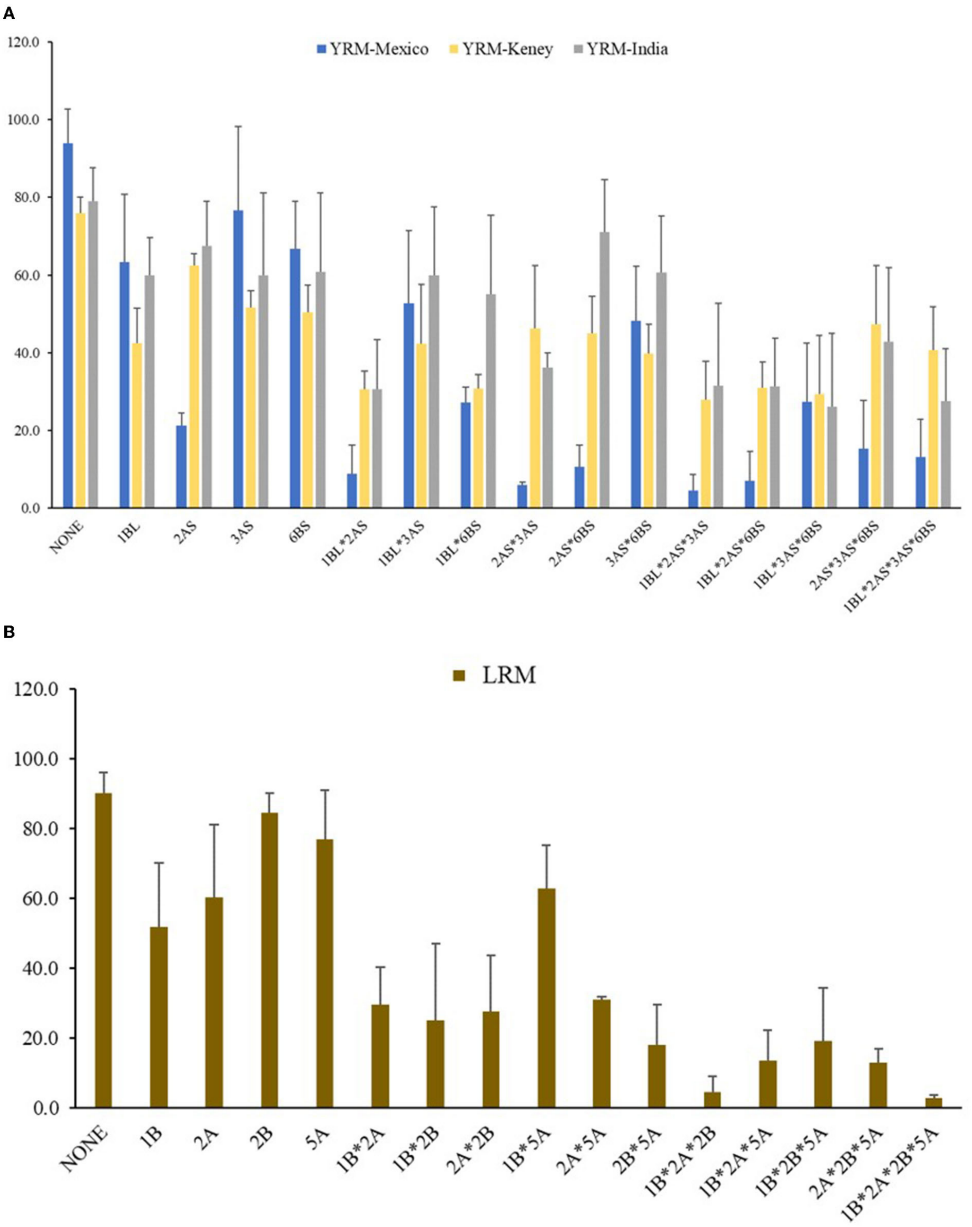
Mean stripe rust severity in three counties **(A)** and leaf rust severity in Mexico **(B)** for lines carrying different QTL combinations, based on the flanking molecular marker of each identified locus from the Apav#1× Mucuy F_5_ RIL population.

The F_5_ RILs were divided into 16 groups according to the genotypes of the 4 stable YR resistance QTL on chromosomes 1Bl, 2AS, 3AS, and 6BS derived from Mucuy. The presence of resistance alleles for the QTL in each RIL was inferred with the QTL combination based on the flanking molecular markers. In Mexico, the disease severity of lines with *QYr.cim-2AS* ranged from 4.5 to 21.3% (Figure 4A). In addition, *QYr.cim-1BL* played a great role in reducing YR severity: the mean DS of YR was 63.3% with *QYr.cim-1BL* present alone, whereas YR DS ranged from 4.5 to 52.7% when *QYr.cim-1BL* was present with other QTL (Figure 4A). Although the resistance effects of *QYr.cim-3AS* and *QYr.cim-6BS* were not significantly different from lines without any resistance QTL, they conferred a significantly lower YR DS when combined with *QYr.cim-2AS* or *QYr.cim-1BL*. In Kenya and India (Figure 4A), *QYr.cim-1BL* showed a higher effect than *QYr.cim-2AS*, when both were present alone but the presence of both loci reduced DS in the line, overall, with the highest average DS of 40.6%.

The F_5_ RILs were classified into 16 groups according to the genotype of 4 LR resistance QTL (Figure 4B). When present alone, *QLr.cim-1BL, QLr.cim-2AS, QLr.cim-2BS* and *QLr.cim-5AL* reduced the LR DS from 90.0% to 51.9, 60.3, 84.4, and 76.9%, respectively. Although the resistance effect provided by *QLr.cim-2BS* alone was small, it had a significant additive effect when combined with other loci. The DS ranged from 2.8 to 27.5% when *QLr.cim-2BS* was present with other QTL. In general, the number of QTL was negatively correlated with DS for RILs.

## Discussion

Mucuy was highly resistant to YR in multi-year field trials in Mexico, Kenya, and India. Resistance in seedlings to Mexican *Pst* isolates and *Pt* races was based on *YrMu* and *Lr16*. In total, our molecular mapping identified 4 LR and 7 YR resistance loci. These loci together explained 74.4% of LR phenotypic variation at the adult plant stage, and 84.2, 43.2, and 58.9% of YR phenotypic variation for Mexican, Kenyan, and Indian *Pst* populations and field environments, respectively. All resistance loci/genes were derived from Mucuy, apart from *QYr.cim-1AL*. The newly developed molecular markers for the 2 co-located resistance loci will help wheat breeders to develop new varieties with more durable resistance to rusts.

### Resistance loci on group 1 chromosomes

So far, chromosome 1AL lacks a formally designated *Yr* gene. In this study, the only resistance locus derived from susceptible parent Apav#1, *QYr.cim-1AL*, explained 2.2–5.4% of the YR phenotypic variation in Mexican environments and 3.8–14.6% of the YR phenotypic variation in Kenyan environments; however, no YR resistance conferred by this locus was detected in Indian environments, suggesting that *QYr.cim-1AL* provides either small race-specific resistance or is environmentally unstable. The physical location range of *QYr.cim-1AL* on the CS reference genome was 565.5–580.0 Mb (International Wheat Genome Sequencing Consortium, 2018). Several QTL have been reported near this interval (Ren et al., 2012a; Rosewarne et al., 2012; Basnet et al., 2014b). Thus, *QYr.cim-1AL* might be the same YR resistance locus from Pastor, Naxos, and TAM112, based on their physical positions of the CS reference genome (International Wheat Genome Sequencing Consortium, 2018). This needs to be further confirmed through gene cloning.

A co-located resistance locus *QYr.cim-1BL/QLr.cim-1BL* was identified on the long arm of wheat chromosome 1B. *QYr.cim-1BL/QLr.cim-1BL* showed stable and significant resistance effects in all tested environments for both YR and LR. We genotyped the RIL population with *cslv46G22*, a closely linked molecular marker for *Lr46/Yr29/Sr58/Pm39* located on 1BL, which showed that *cslv46G22* was loosely linked with *QYr.cim-1BL/QLr.cim-1BL* as one of the flanking markers. To further verify the relationship among *QYr.cim-1BL/QLr.cim-1BL* and *Lr46/Yr29/Sr58/Pm39*, we removed the effect of *cslv46G22* and re-did the QTL analysis but were unable to detect any other resistance locus on 1BL. Therefore, we conclude that *QYr.cim-1BL/QLr.cim-1BL* should be the known APR gene *Lr46/Yr29/Sr58/Pm39*. Over the last two decades, many CIMMYT derived bread and durum wheat have been reported to possess *Lr46/Yr29/Sr58/Pm39*: for example, “Pavon 76” (William et al., 2006), “Saar” (Lillemo et al., 2008), “Pastor” (Rosewarne et al., 2012), “Quaiu 3” (Basnet et al., 2013), “Francolin#1” (Lan et al., 2014), “Sujata” (Lan et al., 2015), “Kundan” (Ren et al., 2017), “Bairds” (Lan et al., 2017b), “Chilero” (Ponce-Molina et al., 2018), and Arableu#1 (Yuan et al., 2020). Based on all reported studies, *Lr46/Yr29/Sr58/Pm39* should be placed from 662.1 to 684.8 Mb, according to the CS reference genome (International Wheat Genome Sequencing Consortium, 2018). The relatively diffused localization of *Lr46/Yr29/Sr58/Pm39* might be due either to the genetic background effect, phenotyping errors, different genotyping platforms, or population size. In addition, recent studies have reported that more than one pleiotropic APR locus could be present in the 1BL region (Yuan et al., 2020; Zhou et al., 2021), indicating that the 1BL region might carry a gene cluster composed of multiple APR genes, which will be confirmed by future gene cloning.

### Resistance loci on group 2 chromosomes

Another co-located resistance locus, *QYr.cim-2AS*/*QLr.cim-2AS*, and the seedling YR resistance gene *YrMu* were located on 2AS (Supplementary Tables 5, 6). *Yr17* is located on a translocation on wheat chromosome 2AS derived from the *Aegilops ventricosa* 2NvS segment, which is known to confer resistance against multiple wheat diseases but also plays role in increasing wheat yields (Gao et al., 2021) and has been used in CIMMYT wheat breeding. This translocation was initially reported to provide significant resistance to all three rusts due to the loci of *Yr17, Lr37*, and *Sr38* (Bariana and McIntosh, 1993; Chen, 2005). However, races virulent to *Yr17* rapidly evolved, once this gene was deployed in Europe (Bayles et al., 2000), Mexico (Randhawa et al., 2018), and in the Indian *Pst* population, due to the widespread cultivation of the *Yr17*-carrying variety HD2967 during the mid-2010's (unpublished results). However, we detected significant YR resistance on 2AS that was stable over 3 years in Mexico against the *Pst* isolates used in phenotyping, and this resistance was also identified in Kenya in 2016. PstS1 was an invasive strain that originated in East Africa in the early 1980's; PstS2 evolved from PstS1 and the two strains have become the dominant races in East Africa (Walter et al., 2016). As of 2019, the new genetic group PstS11 was reported as dominant in East Africa by the Global Rust Reference Center and, unlike PstS1 or PstS2, PstS11 is virulent to *Yr17*. Several reports have indicated that *Yr17* might be considered a race-specific APR gene due to the difficulty and inconsistent seedling phenotypes, especially with the aggressive Pst1 lineage (Fang et al., 2011; Milus et al., 2015). In the present study, the seedling reaction of Mucuy was very low, while the single gene line of *Yr17* (Avocet+*Yr17*) was susceptible to the Mexican *Pst* isolate Mex.14.191. However, *YrMu* was mapped on wheat chromosome 2AS at 16.6–19.1 Mb (International Wheat Genome Sequencing Consortium, 2018) and it co-segregated with 79 molecular markers including *InD_hzau_MuYLr-2AS, WGGB156*, and *WGGB159*, the latter of which were closely linked to *Yr17* (Wang et al., 2018). Thus, *YrMu* should be the known YR resistance gene *Yr17* and the enhanced expression of *Yr17* in Mucuy seedlings might be due to the background effect of other APR loci, or due to the residual effect of the ineffective *Yr17* on APR. Similarly, an LR APR QTL was also located on the translocation, in addition to *Lr37*, because *Pt* races MCJ/SP and MBJ/SP used in field trials are known to be virulent to *Lr37* (Huerta-Espino et al., 2011).

Several previous studies have shown that chromosome 2BS possesses various race-specific and quantitative resistance loci to LR (Messmer et al., 2000; Xu et al., 2005). So far, six formally named LR resistance genes have been identified on chromosome 2BS, including *Lr13* (Seyfarth et al., 1999), *Lr16* (McCartney et al., 2005), *Lr23* (Datta et al., 2008), *Lr35* (Seyfarth et al., 1999)*, Lr48* (Bansal et al., 2008), and *Lr73* (Park et al., 2014). In this study, polymorphism was found in both parents and RILs for *Xgwm210*, the closely linked molecular markers of *Lr16* and the seedling reaction for Mucuy were similar to that of Thatcher+*Lr16* against *Pt* races MBJ/SP. So, by comparing the physical locations, *QLr.cim-2BS* and *Lr16* should be the same gene that conferred LR resistance in Mucuy. *Lr16* was a widely deployed LR resistance gene and conferred moderate resistance at both seedling and adult plant stages, and also showed high additive effects with other resistance genes in the field (Lan et al., 2014), which can be considered useful for breeding.

### Resistance loci on group 3 chromosomes

*QYr.cim-3AS* was detected in both Mexico and Kenya for multiple years, but not in India. Therefore, it was also considered an unstable or race-specific APR gene. So far, only one formally named gene, *Yr76* (Xiang et al., 2016), is mapped on 3AS, but *QYr.cim-3AS* should be distinct from *Yr76*, given that the latter was a seedling resistance gene. For similar reasons, *YrEDWL* (Liu et al., 2017) and several YR resistance-linked SNPs (Jighly et al., 2015) were identified on 3AS but are unlikely to be *QYr.cim-3AS*. In addition, two APR QTL have been mapped on 3AS in the CIMMYT wheat line “Saar” (Lillemo et al., 2008) and the Swiss winter wheat cultivar Arina (Buerstmayr et al., 2014). *QYr.cim-3AS* might be the same as the two QTLs, according to its physical position based on the CS reference genome (International Wheat Genome Sequencing Consortium, 2018).

There are 4 officially named genes on 3BS: *Yr4* (Bansal et al., 2010), *Yr30* (Singh et al., 2000b), *Yr57* (Randhawa et al., 2015), and *Yr58* (Chhetri et al., 2016). *QYr.cim-3BS* should be different from *Yr4* and *Yr57* because the latter two provide resistance at the seedling stage and their physical positions are around 3.3 Mb (*Xbarc75*), which is at least 7.3 Mb away from *QYr.cim-3BS*. *Yr58* starts to express and confer resistance at the four-leaf stage. Although *Yr30* is a common APR gene in CIMMYT materials, the approximate physical interval of this gene is in the telomeric region distal to 10 Mb (William et al., 2006; Rosewarne et al., 2012; Basnet et al., 2014a; Lan et al., 2014; Wu et al., 2017; Jia et al., 2020), but *QYr.cim-3BS* is positioned at 10.6–24.9 Mb. Other YR resistance QTL located on 3BS, considered distinct from *Yr30*, include *QYrhm.nwafu-3BS* (Yuan et al., 2018), *QYrsk.wgp-3BS* (Liu et al., 2019), and *Qyrto.swust-3BS* (Zhou et al., 2019). The physical locations of *QYrhm.nwafu-3BS* and *QYrsk.wgp-3BS* overlapped with *QYr.cim-3BS*; it is possible that they are the same gene/allele, but this needs further verification. It seems that there is more than one APR gene with significant resistance to YR in the 3BS region, other than just *Yr30*, but we cannot rule out the possibility of the presence of *Yr30* in Mucuy, since *Yr30* is not cloned yet.

*QYr.cim-3DS* was identified only in Kenya for 2 consecutive years and accounted for 3.6–7.7% of the phenotypic variation. Singh et al. (2000b) and Boukhatem et al. (2002) both mapped a QTL on 3DS in the CIMMYT wheat “Opata 85.” The tightly linked marker, *Xbcd532*, is physically located at least 37.1 Mb away from *QYr.cim-3DS*. There are several other YR resistance genes identified on chromosome 3DS, such as *Yr49* (McIntosh et al., 2017), *Yr66* (McIntosh et al., 2013), *YrY206* (Zhang et al., 2008), *QYr.inra-3DS* (Dedryver et al., 2009), *YrS1* (Sun et al., 2019), and *QYrsn.nwafu-3DS* (Huang et al., 2020). Among them, *QYrsn.nwafu-3DS* provides resistance only in seedlings and *YrY206* originated from *Aegilops tauschii* (Coss.) *Schmal*. According to the physical location of the flanking markers (International Wheat Genome Sequencing Consortium, 2018), *Yr49* (*Xgwm161*) and *YrS1* (*Xcfd79*) were mapped 71.7 and 65.6 Mb distal to *QYr.cim-3DS*, respectively. *Yr66* (*Xgwm341*) and *QYr.inra-3DS* (*Xgwm456*) are at least 18.8 and 122.9 Mb away from *QYr.cim-3DS*, respectively. Based on the source of resistance, resistance characteristics, and physical location comparisons, *QYr.cim-3DS* might be a new YR APR QTL, in Kenyan environments.

### Resistance loci on group 5 chromosomes

*QLr.cim-5AL* was located on 5AL, where no other LR resistance gene has been officially designated. Rosewarne et al. (2012) detected a QTL on 5AL derived from Avocet that provided APR to LR. The LOD peak of this locus was near *wPt-0837*, which corresponds to a physical location of 621.6 Mb, based on the CS reference genome (International Wheat Genome Sequencing Consortium, 2018). *QLr.cim-5AL* detected in the present study was located on 585.2–589.2 Mb and was derived from Mucuy, so the two QTL should be different, given the ~35 Mb gap between them. Recently, Zhang et al. (2019) identified *QLr.hebau-5AL* flanked by *AX-110679506* and *AX-110996595*, which corresponded to 589.3–591.4 Mb of the CS reference genome (International Wheat Genome Sequencing Consortium, 2018). This locus explained 6.6–7.1% of the variation in the LR resistance response and was derived from a resistant cultivar SW 8588, whose pedigree includes the CIMMYT variety Milan. Therefore, this locus is likely to be *QLr.cim-5AL*. However, *QLr.hebau-5AL* also had an effect on YR across four environments in China, whereas no effect of *QLr.cim-5AL* on YR was identified in the present study. We speculate that this could be due to different *Pst* isolates present in China and Mexico or a genetic background effect.

### Resistance loci on group 6 chromosomes

*QYr.cim-6BS* was identified on 6BS. There are three named YR resistance genes on this chromosome: *Yr35* (Marais et al., 2003), *Yr36* (Uauy et al., 2005), and *Yr78* (Dong et al., 2017). *Yr35* provides all-stage resistance and was transferred to wheat from *T. turgidum* ssp. *dicoccoides*. *Yr36* also originated from *T. dicoccoides* and encodes a kinase-START protein that confers temperature-dependent broad-spectrum resistance (Fu et al., 2009). *QYr.cim-6BS* is unlikely to be *Yr35* or *Yr36*, based on the source and its resistance characteristics. *Yr78* provides APR only in the field (Dong et al., 2017). Comparing the genetic distance and physical position between the corresponding closely linked molecular markers (Somers et al., 2004), several QTL providing APR to YR that map on 6BS could likely be *Yr78*. These include *QYrst.wgp-6BS.1* (Santra et al., 2008), *QYr.inra-6B* (Dedryver et al., 2009), *QYr.caas-6BS* (Lan et al., 2010), *QYr.caas-6BS.3* (Ren et al., 2012b), and *QYrMA.wgp-6BS* (Liu et al., 2018). The physical locations of several markers closely linked to *Yr78* are included in the range of *QYr.cim-6BS* detected in this study, such as *Xwmc104* (149.1 Mb) and *Xbarc136* (151.3 Mb). Therefore, it is likely that *QYr.cim-6BS* is the same as *Yr78*, but further verification of this is needed.

The evolution of new virulence and pathogen migration and adaptation to unconventional environments has been observed in the last decade. In addition to the rapid mutation from avirulence to virulence in rust fungi, global climate change and the limited use of resistance genes in complex combinations are important contributors. The average effective life of a race-specific resistance gene is 2–4 years, with the evolution of new races in Mexico. Breeding new varieties with durable resistance is the most effective way to control wheat diseases. Mucuy was distributed for international testing in 2013 and showed high resistance to both YR and LR in Mexico, India, Kenya, and China, suggesting that is a good choice as the donor for introducing resistance into other elite breeding materials. The new resistance loci identified in our study can be further studied to characterize their effects and interactions in other genetic backgrounds and thereby derive the best combinations of effective resistance genes to enhance durability.

## Data availability statement

The original contributions presented in the study are included in the article/Supplementary material, further inquiries can be directed to the corresponding authors.

## Author contributions

RS, CY, JH-E, MR, SB, EL, and CL conceived of the project. DL, CY, and CL wrote the article, performed QTL mapping, and SSR and KASP analyses. RS and CL performed the developmental analyses. JH-E, MR, SB, and EL evaluated the phenotyping of the RILs. All authors contributed to the article and approved the submitted version.

## Funding

This study was supported by International Cooperation and Exchange of the National Natural Science Foundation of China (Grant Nos. 31861143010 and 32101712), Hubei Hongshan Laboratory (Grant Nos. 2022hspy001 and 2021hskf008), the Strategic Priority Research Program of the Chinese Academy of Sciences (Grant No. XDA24030102), Construction Project for Innovation Platform of Qinghai Province (Grant No. 2022-ZJ-Y04), Construction Project for Innovation Platform of Qinghai Province (Grant No. 2022-ZJ-Y01), Key R & D and Transformation Program of Qinghai Province (Grant No. 2022-NK-106), the Australian Grains Research and Development Corporation (GRDC) with funding to the Australian Cereal Rust Control Program (ACRCP), CGIAR Research Program WHEAT (CRPWHEAT), and the Foundation of Application of Basic Research Project of Qinghai Province (Grant No. 2022-ZJ-737).

## Conflict of interest

The authors declare that the research was conducted in the absence of any commercial or financial relationships that could be construed as a potential conflict of interest.

## Publisher's note

All claims expressed in this article are solely those of the authors and do not necessarily represent those of their affiliated organizations, or those of the publisher, the editors and the reviewers. Any product that may be evaluated in this article, or claim that may be made by its manufacturer, is not guaranteed or endorsed by the publisher.

## Supplementary material

Supplementary Figure 1 Genetic linkage maps of stripe rust (YR) seedling resistance gene *YrMu* on the chromosome 2NS/2AS **(A)** and of leaf rust (LR) seedling resistance gene *Lr16* on wheat chromosome 2BS **(B)**, after removing redundant markers. Locus names and corresponding locations on the genetic map are indicated on the right side. Map distances in cM are shown on the left side.

Supplementary Figure 2 Scatter plots for KASP marker *Kasp_hzau_MuYLr-1BL* genotyping in the “Apav#1 × Mucuy” F_5_ RIL population.

Supplementary Table 1 Avirulence/virulence information for rust races used at Ludhiana, India. *Yrso*, Suwon92Omar; *Yrsp*, Spalding Prolific; *Yrsk*, Selkirk; *Yrsd*, Strubes Dickkopf; *Yrks*, Kalyansona.

Supplementary Table 2 Sequences of newly developed primers used to detect the co-located resistance loci *QYr.cim-1BL/QLr.cim-1BL* and *QYr.cim-2AS/QLr.cim-2AS*.

Supplementary Table 3 Single marker analysis of KASP marker *Kasp_hzau_MuYLr-1BL*.

Supplementary Table 4 Single marker analysis of InDel marker *InD_hzau_MuYLr-2AS*.

Supplementary Table 5 The physical position of *QYr.cim-2AS/YrMU/QLr.cim-2AS*'s flanking marker based on reference genomes of Chinese Spring and Jagger.

Supplementary Table 6 The physical location of *QYr.cim-2AS*'s flanking marker showed that the actual genome sequence of Mucuy on Chromosome 2AS was different from that of Chinese Spring.
