# Supplementary figures and images for: Stripe rust and leaf rust resistance in CIMMYT wheat line “Mucuy” is conferred by combinations of race-specific and adult-plant resistance loci
Source: Front Plant Sci. 2022 Aug 19;13:880138. doi: 10.3389/fpls.2022.880138 (PMC9437451; doi:10.3389/fpls.2022.880138)

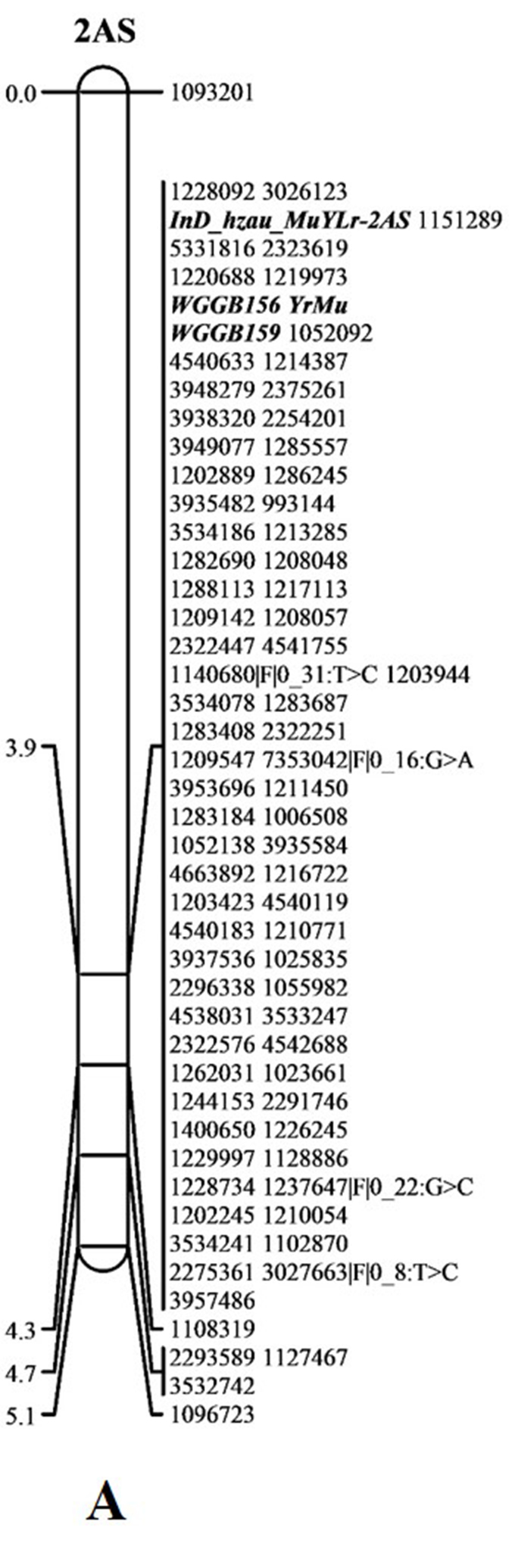

Supplement: Supplementary Figure 1 — Genetic linkage maps of stripe rust (YR) seedling resistance gene YrMu on the chromosome 2NS/2AS (A) and of leaf rust (LR) seedling resistance gene Lr16 on wheat chromosome 2BS (B), after removing redundant markers. Locus names and corresponding locations on the genetic map are indicated on the right side. Map distances in cM are shown on the left side. [file Image_1.JPEG]

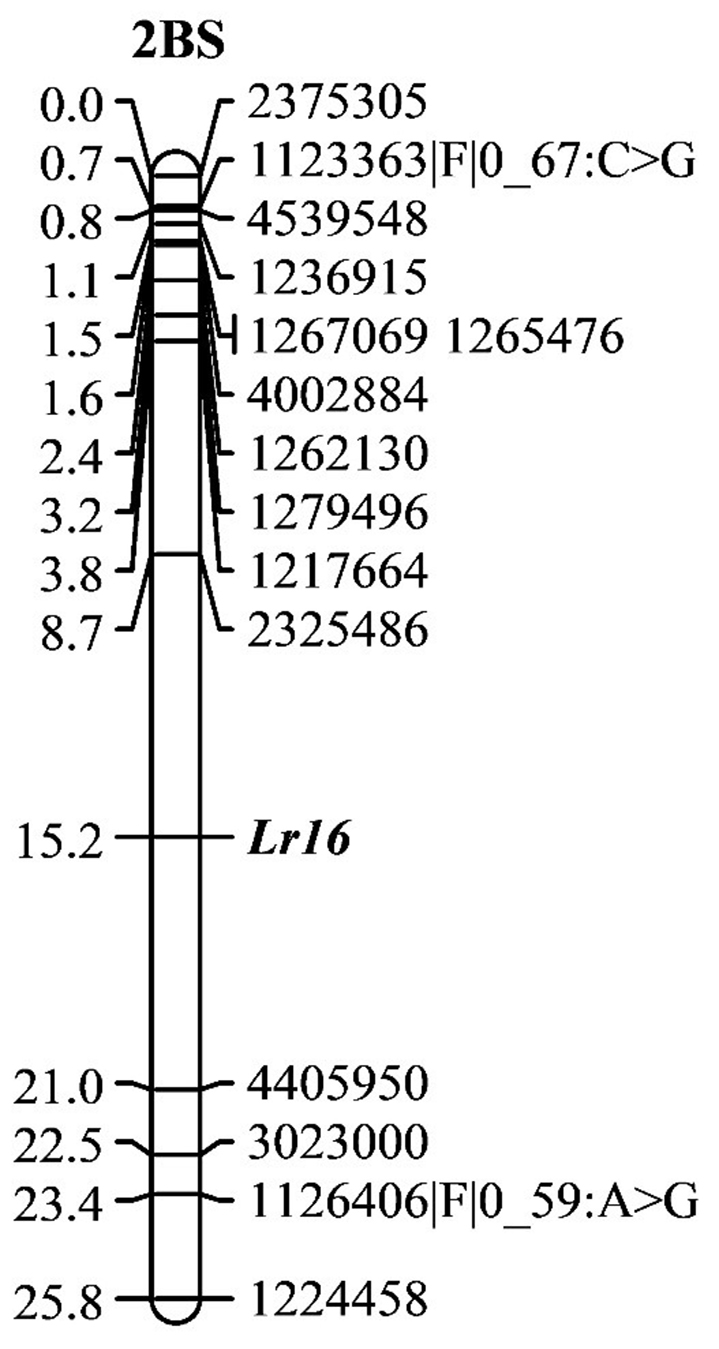

Supplement: Supplementary Figure 2 — Scatter plots for KASP marker Kasp_hzau_MuYLr-1BL genotyping in the “Apav#1 × Mucuy” F5 RIL population. [file Image_2.JPEG]

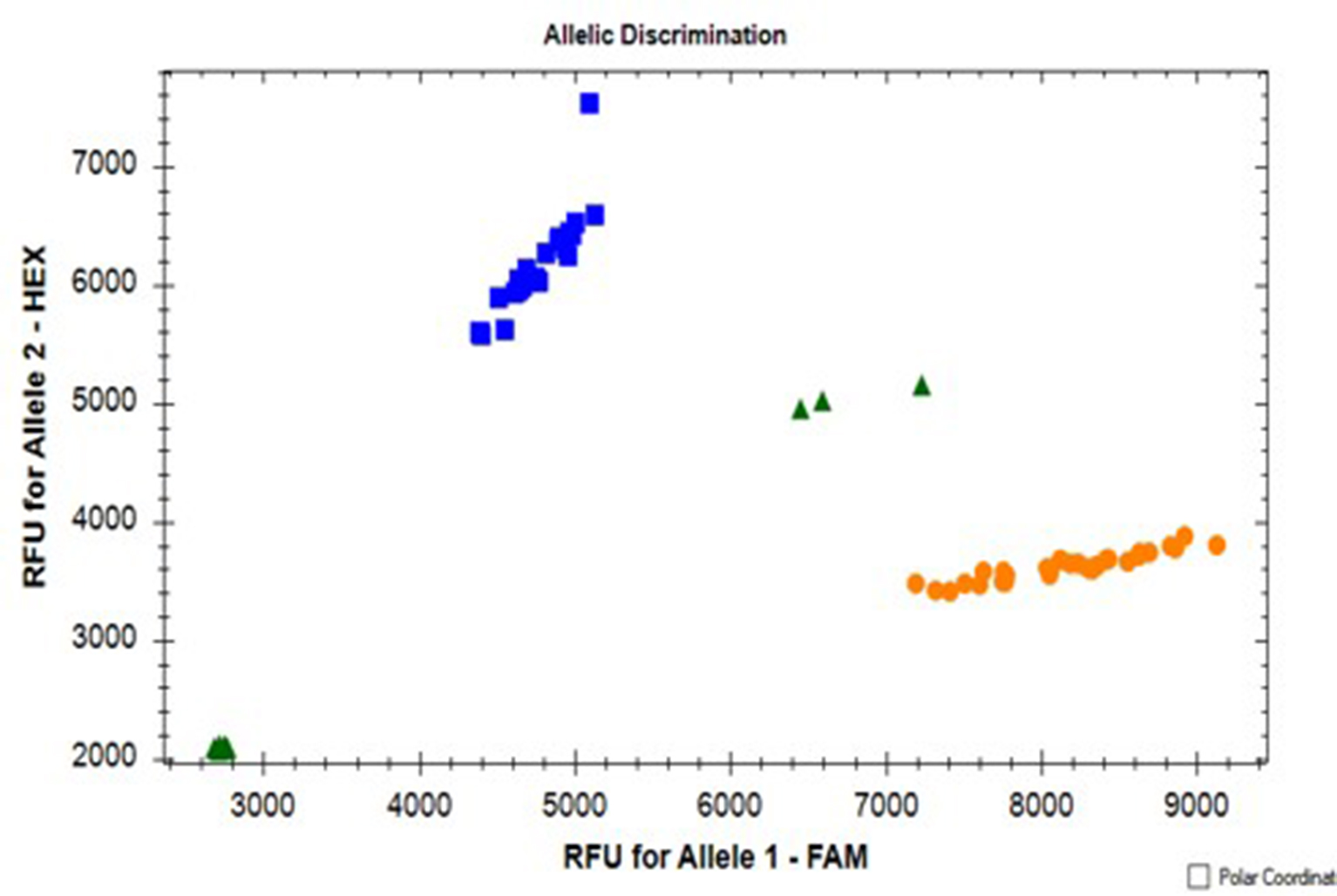

Supplement: Supplementary file 4 [file Image_3.JPEG]
