# Supplementary material for: Stripe rust and leaf rust resistance in CIMMYT wheat line “Mucuy” is conferred by combinations of race-specific and adult-plant resistance loci
Source: Front Plant Sci. 2022 Aug 19;13:880138. doi: 10.3389/fpls.2022.880138 (PMC9437451; doi:10.3389/fpls.2022.880138)
Supplement: Supplementary Table 1 — Avirulence/virulence information for rust races used at Ludhiana, India. Yrso, Suwon92Omar; Yrsp, Spalding Prolific; Yrsk, Selkirk; Yrsd, Strubes Dickkopf; Yrks, Kalyansona. [file Data_Sheet_1.docx]

**Supplemental Table 1.** Avirulence/virulence information for rust races used at Ludhiana, India.

| Sno. | Pt. | Avir. | Vir. |
| --- | --- | --- | --- |
| 1 | 46S119 | *Yr1, Yr5, Yr10, Yr11, Yr12, Yr13, Yr14, Yr15, Yr16, Yr24, Yr26, Yrsp, Yrso , Yrsk* | *Yr2, Yr3, Yr4, Yr6, Yr7, Yr8, Yr9, Yr17, Yr18, Yr19, Yr21, Yr22, Yr23, Yr25, YrA, Yrsd, Yrso* |
| 2 | 110S84 | *Yr1*, *Yr2*, *Yr5*, *Yr2, Yr10*, *Yr11, Yr12, Yr13, Yr14*, *Yr15, Yr16, Yr24* *, Yr26, Yrsp, Yrsk* | *Yr2Ks*,*Yr4, Yr6*, *Yr8*, *Yr9*, *Yr7*, *YrSD,* *Yrso* |
| 3 | 110S119 | *Yr1*, *Yr2*, *Yr5*, *Yr10*, *Yr11, Yr12, Yr13, Yr15, Yr16, Yr24, Yrsp, Yrsk* | *Yr2*, *Yr3, Yr4*, *Yr6*, *Yr7*, *Yr8, Yr9* , *Yr14, YrSD*, *Yrso* |
| 4 | 238S119 | *Yr1*, *Yr5*, *Yr10*, *Yr11, Yr12, Yr13, Yr14*, *Yr15, Yr16,* *Yr24,* *Yrsk* | *Yr2*, *Yr3*, *Yr4*, *Yr6*, *Yr7*, *Yr8*, *Yr9*, *Yr9* (Riebesel47/51), *YrSD,* *YrSo* |

*Yrso*=Suwon92Omar, *Yrsp*=Spalding Prolific, *Yrsk*=Selkirk, *Yrsd*=Strubes Dickkopf, *Yrks*= Kalyansona

**Supplemental Table 2.** Sequences of newly developed primers used to detect the co-located resistance loci *QYr.cim-1BL/QLr.cim-1BL* and *QYr.cim-2AS/QLr.cim-2AS*.

| Primer | Sequences |
| --- | --- |
| *Kasp_hzau_MuYLr-1BL* *-A* | GAAGGTGACCAAGTTCATGCTtgcaaatccactcgattgcT |
| *Kasp_hzau_MuYLr-1BL* *-B* | GAAGGTCGGAGTCAACGGATTtgcaaatccactcgattgcC |
| *Kasp_hzau_MuYLr-1BL-Common* | gcaattctcgttcagcagcc |
| *InD_hzau_MuYLr-2AS -F* | CGATCCTTCTGAATCCACATCA |
| *InD_hzau_MuYLr-2AS -R* | TTGACCAATGAAACCGTTACCA |

**Supplemental Table 3.** Single marker analysis of KASP marker *Kasp_hzau_MuYLr-1BL*.

| Environment | Mu-1BL | _Mu-1BL | Estimate ^a^ | Error | DF^b^ | *t* Value | *Pr* > \|t\| |
| --- | --- | --- | --- | --- | --- | --- | --- |
| YrMV15 | A^c^ | B^d^ | 15.6 | 5.0 | 135 | 3.1 | 0.0025 |
| YrMV15AU | A | B | 107.0 | 33.2 | 135 | 3.2 | 0.0016 |
| YrMV16 | A | B | 24.5 | 5.7 | 135 | 4.3 | <.0001 |
| YrMV16AU | A | B | 266.7 | 63.3 | 130 | 4.2 | <.0001 |
| YrMV17 | A | B | 20.9 | 5.1 | 135 | 4.1 | <.0001 |
| YrMV17AU | A | B | 155.1 | 45.5 | 135 | 3.4 | 0.0009 |
| YrKE16O | A | B | 33.9 | 4.4 | 134 | 7.7 | <.0001 |
| YrKE16M | A | B | 22.6 | 3.0 | 135 | 7.4 | <.0001 |
| YrKE19 | A | B | 15.3 | 4.0 | 135 | 3.9 | 0.0002 |
| YrKE20 | A | B | 10.9 | 2.6 | 135 | 4.2 | <.0001 |
| YrIN19 | A | B | 16.3 | 3.5 | 135 | 4.7 | <.0001 |
| YrIN20 | A | B | 24.4 | 5.5 | 135 | 4.5 | <.0001 |
| YrM | A | B | 19.9 | 3.1 | 135 | 6.4 | <.0001 |
| LrY16 | A | B | 22.2 | 4.3 | 135 | 5.2 | <.0001 |
| LrY16AU | A | B | 213.1 | 38.5 | 135 | 5.5 | <.0001 |
| LrY17 | A | B | 41.8 | 5.3 | 135 | 7.9 | <.0001 |
| LrY17AU | A | B | 395.1 | 54.2 | 135 | 7.3 | <.0001 |
| LrY18 | A | B | 37.0 | 5.8 | 135 | 6.4 | <.0001 |
| LrY18AU | A | B | 252.8 | 37.1 | 135 | 6.8 | <.0001 |
| LrM | A | B | 33.6 | 4.9 | 135 | 6.9 | <.0001 |

^a^ Mean phenotypic differences between A and B genotypes in different environments.

^b^ means the degree of freedom

^c^ means the absent of the allele

^d^ means the present of the allele

**Supplemental Table 4.** Single marker analysis of InDel marker *InD_hzau_MuYLr-2AS*.

| Environment | Mu-2AS | _Mu-2AS | Estimate ^a^ | Error | DF^b^ | *t* Value | *Pr* > \|t\| |
| --- | --- | --- | --- | --- | --- | --- | --- |
| YrMV15 | A^c^ | B^d^ | 42.7 | 3.6 | 136 | 12.0 | <.0001 |
| YrMV15AU | A | B | 293.1 | 22.5 | 136 | 13.0 | <.0001 |
| YrMV16 | A | B | 50.5 | 4.1 | 136 | 12.4 | <.0001 |
| YrMV16AU | A | B | 574.2 | 42.5 | 131 | 13.5 | <.0001 |
| YrMV17 | A | B | 42.6 | 3.8 | 136 | 11.1 | <.0001 |
| YrMV17AU | A | B | 354.1 | 35.1 | 136 | 10.1 | <.0001 |
| YrKE16O | A | B | 12.9 | 5.1 | 135 | 2.6 | 0.0117 |
| YrKE16M | A | B | 15.0 | 3.3 | 136 | 4.6 | <.0001 |
| YrKE19 | A | B | -2.9 | 4.1 | 136 | -0.7 | 0.4812 |
| YrKE20 | A | B | -0.3 | 2.7 | 136 | -0.1 | 0.9134 |
| YrIN19 | A | B | 3.4 | 3.6 | 136 | 0.9 | 0.3577 |
| YrIN20 | A | B | 21.3 | 5.4 | 136 | 3.9 | 0.0001 |
| YrM | A | B | 21.1 | 3.0 | 136 | 7.1 | <.0001 |
| LrY16 | A | B | 25.9 | 4.0 | 136 | 6.5 | <.0001 |
| LrY16AU | A | B | 245.6 | 36.1 | 136 | 6.8 | <.0001 |
| LrY17 | A | B | 32.3 | 5.6 | 136 | 5.7 | <.0001 |
| LrY17AU | A | B | 328.1 | 56.0 | 136 | 5.9 | <.0001 |
| LrY18 | A | B | 32.7 | 5.8 | 136 | 5.6 | <.0001 |
| LrY18AU | A | B | 223.3 | 37.5 | 136 | 6.0 | <.0001 |
| LrM | A | B | 30.3 | 4.9 | 136 | 6.2 | <.0001 |

^a^ Mean phenotypic differences between A and B genotypes in different environments.

^b^ means the degree of freedom

^c^ means the absent of the allele

^d^ means the present of the allele

**Supplementary Table 5.** The physical position of *QYr.cim-2AS/YrMU/QLr.cim-2AS*’s flanking marker based on reference genomes of Chinese Spring and Jagger.

| Locus | Marker | Sequence | Physical position (CS) | | Physical position (Jagger) | |
| --- | --- | --- | --- | --- | --- | --- |
| *QYr.cim-2AS* | 1208841 | TGCAGTTGGGCAACATCATGATCGCCAACATCACATTGGAGACGGCGCAGATGGTGGTCTACACCTTCC | | 31,970,764-31,970,819 | | 24,816,691-24,816,759 |
| *QYr.cim-2AS* | 978751 | TGCAGGCACCATTGCTGATATGCGGCAGCATTCGGAGTGGTATTATGACAAGCATTATGTTGTACTGCC | | 32,821,281-32,821,349 | | 41,102,750-41,102,682 |
| YrMU | WGGB156 | F: TATGTCAGTGAGCCGCCTAG | | 16,602,089-16,602,480 | | 22,084,552-22,084,533 |
|  |  | R: AACACTGGGAGGTAAGCTCC | |  |  |  |
| YrMU | WGGB159 | F: CGGTGCTATTGGTCGTTCAG | | 19,122,125-19,122,528 | | No hits found |
|  |  | R: TCCATCTTAGGTTGCTGGCA | |  |  |  |
| QLr.cim-2AS | 100033379\|F\|0_5:A>G | TGCAGACCAAGGCGATCCTGACTGGCCCGAGATCGGAAGAGCGGTTCAGCAGGAATGCCGAGACCGATC | | 4,942,954-4,942,926 | | 7,395,918-7,395,890 |
| QLr.cim-2AS | 3952334 | TGCAGGTCTATGATGCATTCTTCCGCCACCTTCGACTGCGCCGATTCTTCCTTGACATCTACAAGCTCC | | 19,447,986-19,447,918 | | 28,128,771-28,128,703 |
| QLr.cim-2AS | 997868 | TGCAGAGAGCGATAGAATCTGGTGGTGGTGCATTGGGGGATGGGGGTGAAGTGGATGGATGATGGACAT | | 15,355,496-15,355,428 | | 20,027,254-20,027,196 |
| QLr.cim-2AS | 1085721 | TGCAGCGGAGCGAGATTGTCAGGCTCGGCCGAGGAGGTGACGGCGCCCCGTCGTCGTTGGCCCCCGAGA | | 15,467,466-15,467,531 | | 20,191,939-20,192,007 |

**Supplementary Table 6.** The physical location of *QYr.cim-2AS*’s flanking marker showed that the actual genome sequence of Mucuy on Chromosome 2AS was different from that of Chinese Spring.

| Marker | Genetic position (cM) | Sequence | Physical position (CS) (Mb) |
| --- | --- | --- | --- |
| 4544106 | 118.648 | TGCAGCAGCAGCCCGCTCCACGTCCTCATCTTCCTCCTCCTCCTCATCATCATGCTGCTGCCTACCATG | 35,877,217-35,877,285 |
| 4405948 | 118.686 | TGCAGAACTTTGGCGCTGCATGCAGGTTCCGAGATCGGAAGAGCGGTTCAGCAGGAATGCCGAGACCGA | none |
| 1206392 | 118.686 | TGCAGTTTCAGCGGCCTCGGTGATTTTTCAACGCATGCATGCGTCACTCCGAGATCGGAAGAGCGGTTC | none |
| 2322268 | 118.686 | TGCAGTTCGTCACTCTGACTCTTCGCCCTCGTAGCAGTCGCGGTCCTCATCGATATCACCTTCGAAAGA | 12,199,376-12,199,426 |
| 1010794 | 118.686 | TGCAGGGGGAGATGGGATCGGCTTCGCCGTTCGGTAGTATCTCTTCACCGGCTCATAAAGGTCATGAGA | 9,386,978-9,387,046 |
| 1201081 | 118.686 | TGCAGTATATATACCGAGAGTGATGATCTAGGGGCATGTAAGCTCACACTTTCTCACTATACATCTGGG | 11,837,902-11,837,830 |
| 4539219 | 118.748 | TGCAGACGTTTAGCAGGTCAATTTTGCCTTCTCCGAGATCGGAAGAGCGGTTCAGCAGGAATGCCGAGA | none |
| 1205315 | 118.808 | TGCAGAACCCCAGGCAAGACCGCAGGTGCTGAGGCAGATTCCTGTAGGTGGACGCGCCGAGATCGGAAG | none |
| 3941426 | 118.868 | TGCAGATGAGCAACATGGTGACGCTCGAGGAGGTGATATCTGGCAGGGATCGACTTACTCTTTGCCACC | 5,692,454-5,692,415 |
| 1202068 | 118.994 | TGCAGGCCCAGATCCCCATCCGCCACCACAATCTAGATCCGTGTCGTCCATCGGGGCTAGCCCGCAGCC | 20,899,117-20,899,085 |
| 1221121 | 119.05 | TGCAGGCTTCCACACAGATGCTGTAACATGATCCCACTCATTACCGAGGAGTATGTCTCATGGTCTGGA | 7,402,587-7,402,520 |
| 1363303 | 119.05 | TGCAGTCGATCACGCCATCTTCCCTGTCTACCCATGTTCTTTGAGGACGAGGACTGTTGCTGCAACGAC | 220,949,176-220,949,210 |
| 1219903 | 119.166 | TGCAGCTTCCGCGAGGAAATTGGCGGGAGATGATGGGCAGCAGAAAGGCGAAATGGTGAAGAAAATGGA | 18,881,837-18,881,905 |
| 4538040 | 119.252 | TGCAGGACAGCAACGCCATGCACGGCAGCGTCGACTGCTGCCAACGCGCCACCACCCTCGCCGAGATCG | 1,686,950-1,686,886 |
| 4542514 | 119.414 | TGCAGGTTAGATACAGTTTTGTTTCCGAGATCGGAAGAGCGGTTCAGCAGGAATGCCGAGACCGATCTC | 12,221,350-12,221,325 |
| 1208841 | 119.414 | TGCAGTTGGGCAACATCATGATCGCCAACATCACATTGGAGACGGCGCAGATGGTGGTCTACACCTTCC | 31,970,764-31,970,819 |
| 978751 | 125.409 | TGCAGGCACCATTGCTGATATGCGGCAGCATTCGGAGTGGTATTATGACAAGCATTATGTTGTACTGCC | 32,821,281-32,821,349 |
| 1264891 | 125.409 | TGCAGCTGCTCTACGGATGCCCTCTCCTCTGTTGGAATCAACCACGTAGTAGAAGTCCTGTCCGAGATC | 21,310,444-21,310,410 |
| 1104152 | 125.411 | TGCAGGTGCCCCCTATATATCCTTGCTTAACACCATGTGCCTAGCATCTCATACGCCGAGATCGGAAGA | 31,840,183-31,840,126 |
| 1048259 | 125.985 | TGCAGCAGCAGCCCGCTCCACGTCCTCATCTTCCTCCTCCTCCTCATCATCATGCTGCTGCCTACCATG | 35,877,217-35,877,285 |
